# Physicochemical and Biological Properties of Gelatin Extracted from Marine Snail *Rapana venosa*

**DOI:** 10.3390/md17100589

**Published:** 2019-10-17

**Authors:** Alexandra Gaspar-Pintiliescu, Laura Mihaela Stefan, Elena Daniela Anton, Daniela Berger, Cristian Matei, Ticuta Negreanu-Pirjol, Lucia Moldovan

**Affiliations:** 1 Departament of Cellular and Molecular Biology, National Institute of R&D for Biological Sciences, 296 Splaiul Independentei, 060031 Bucharest, Romania; alex.gaspar@yahoo.com (A.G.-P.); danielaanton89@gmail.com (E.D.A.); moldovanlc@yahoo.com (L.M.); 2 Faculty of Applied Chemistry and Material Science, University “Politehnica” of Bucharest, 1-7 Gheorghe Polizu street, 011061 Bucharest, Romania; danaberger01@yahoo.com (D.B.); cristi_matei@yahoo.com (C.M.); 3 Faculty of Pharmacy, University “Ovidius” of Constanta, 1 Aleea Universitatii, 900470 Constanta, Romania; ticuta_np@yahoo.com

**Keywords:** gelatin, marine gastropod, Black Sea, acidic and enzymatic extraction, biocompatibility, cytokines

## Abstract

In this study, we aimed to obtain gelatin from the marine snail *Rapana venosa* using acidic and enzymatic extraction methods and to characterize these natural products for cosmetic and pharmaceutical applications. Marine gelatins presented protein values and hydroxyproline content similar to those of commercial mammalian gelatin, but with higher melting temperatures. Their electrophoretic profile and Fourier transform infrared (FTIR) spectra revealed protein and absorption bands situated in the amide region, specific for gelatin molecule. Scanning electron microscopy (SEM) analysis showed significant differences in the structure of the lyophilized samples, depending on the type of gelatin. In vitro studies performed on human keratinocytes showed no cytotoxic effect of acid-extracted gelatin at all tested concentrations and moderate cytotoxicity of enzymatic extracted gelatin at concentrations higher than 0.5 mg/mL. Also, both marine gelatins favored keratinocyte cell adhesion. No irritant potential was recorded as the level of IL-1α and IL-6 proinflammatory cytokines released by HaCaT cells cultivated in the presence of marine gelatins was significantly reduced. Together, these data suggest that marine snails are an alternative source of gelatins with potential use in pharmaceutical and skincare products.

## 1. Introduction

Gelatin is a protein obtained by thermal denaturation of collagen, the main constituent of connective tissue. Being a derivate product of collagen, gelatin has similar structural features and properties [1]. The primary structure of collagen type I consists of two α1-chains and one α2-chain containing the repeating amino acid sequence Gly–X–Y, where X and Y are mainly proline and hydroxyproline [2]. The specific primary structure leads to left-handed helices (secondary structure) and three alpha chains organize into a right-handed triple helix, forming a collagen molecule of 300 nm in length and less than 2 nm in diameter [3]. The denaturation process of collagen implies partial destruction of its tertiary, secondary and, to some extent, its primary structure, resulting in gelatin as a mixture of proteins and polypeptides [4].

Gelatin is extensively used as a natural biomaterial in tissue engineering due to its high biocompatibility, biodegradability, low antigenicity, and ability to stimulate cellular attachment and growth [5]. So far, commercial gelatin is conventionally obtained from mammalian tissues, like skin and bones from bovine, porcine, or caprine species. Recently, there has been great interest in obtaining gelatin from other sources, especially marine organisms, in order to avoid transmitting bovine spongiform encephalopathy or swine flu, as well as for religious reasons [6]. Moreover, byproducts of different fish species [7,8] and other marine sources have been used for gelatin extraction, such as sponges [9,10,11], jellyfishes [12,13], squids [14,15], or snails [16,17].

*Rapana venosa* is a marine snail belonging to the Muricidae family that is rich in proteins, amino acids, sterols, and vitamins [18,19,20]. It is a predatory marine snail that quickly expanded in the Black Sea, having a negative effect on the ecosystem, especially inducing the decline of different species of mussels and mollusks [21]. Besides its nutritional value [18,22], *R. venosa* is also appreciated as a potential source for biotechnological applications. Previous studies have reported that amino acids and lipids extracted from *R. venosa* exhibited wound-healing properties on rat skin burns [18,23]. Recently, Luo et al. [24] obtained protein hydrolysates from *R. venosa* with significant antioxidant activity.

The aim of this study was to extract and characterize gelatin from the soft tissue of *R. venosa* using acidic and enzymatic methods, in order to assess their use in the pharmaceutical and cosmetic fields. Its physicochemical and ultrastructural properties were analyzed and compared to those of commercial pig skin gelatin. In addition, marine gelatins were tested on human keratinocyte cells for their cytocompatibility, cell adhesion capacity, and irritant potential.

## 2. Results and Discussion

### 2.1. Physicochemical and Structural Properties of Marine Gelatins

#### 2.1.1. Yield Extraction and Gelatin Characteristics

In order to obtain gelatin, insoluble native collagen is subjected to thermal hydrolysis, using chemical and enzymatic methods. Both methods are intended to break the inter- and intramolecular crosslinks without cleavage of the peptide bonds, so that the polypeptide chains remain intact [25]. In our study, after the pretreatment of the marine snail soft tissue with NaOH, chemical hydrolysis was performed using acetic acid, an organic acid capable to induce higher solubility of the tissue during the extraction process [25], resulting in an acid-solubilized gelatin (ASG) solution. The pepsin-solubilized gelatin (PSG) solution was obtained by enzymatic hydrolysis with pepsin, which cleaves bonds in the telopeptide region of the collagen structure [26]. Applying these two methods at 60 °C, we have obtained a higher extraction yield for the acidic treatment (9.71%), compared to the enzymatic one (8.65%) (Table 1).

The yield values were comparable to those of gelatin extracted from squids (7.5%), but were lower than those obtained from jellyfish (11.8%–12%) and several fish species (ranging between 11.3% and 20.27%) [14,27,28]. A previous study reported a yield of 8.69%, 10.57%, and 6.54% of gelatin extracted from the body, foot, and viscera of *Ficus variegata* gastropod [16]. The gelatin extraction yields are affected by the tissue used for extraction, the quantity of soluble components in the source, or the collagen content [17]. According to Jamilah and Harvinder [29], the yield extraction can also be influenced by other factors, such as temperature, time of extraction, concentration of NaOH or acetic acid, tissue/enzyme ratio, and pH. The temperatures ranging between 45 and 60 °C are considered optimal regarding the extraction yield of gelatin [30,31]. Higher temperature and longer time of extraction could result in an increased yield, but loss of functional properties of gelatin [30].

The protein content of both marine gelatins was high (91.48% for ASG and 83.12% for PSG) and comparable to that of the commercial pig skin gelatin (CG), indicating the efficiency of the used extraction methods (Table 1).

ASG and CG samples showed similar Hyp values (10.62% and 11.17%, respectively), while PSG exhibited a slightly lower Hyp content (9.39%). Glycine, proline, and hydroxyproline are the most abundant amino acids found in variable amounts in gelatin compositions depending on the source. The total amount of proline and hydroxyproline in fish gelatin is about 16%–20% [32]. Our data were comparable to those reported for gelatin extracted from ribbon jellyfish (*Chrysaora* sp.) and tilapia fish skin, with a Hyp content of 8.2% and 10.31%, respectively [33,34].

The melting temperature, determined by differential scanning calorimetry, was higher for ASG and PSG samples (35.3 and 33.2 °C, respectively) compared to CG (28.8 °C) (Table 1, Supplementary Figure S1). However, the melting temperature values of ASG and PSG extracted from *R. venosa* were close to those reported for gelatin derived from other marine sources, such as *Chondrosia reniformis* (30.48 °C) and *Thymosia guernei* (31.02 °C) marine sponges [35]. In our study, the gelatin samples obtained from the marine snail *R. venosa* showed thermal stability, indicating the possibility of using these components for the development of new biomaterials that require heat resistance.

#### 2.1.2. SDS-Polyacrylamide Gel Electrophoresis (SDS-PAGE)

**Figure 1:**
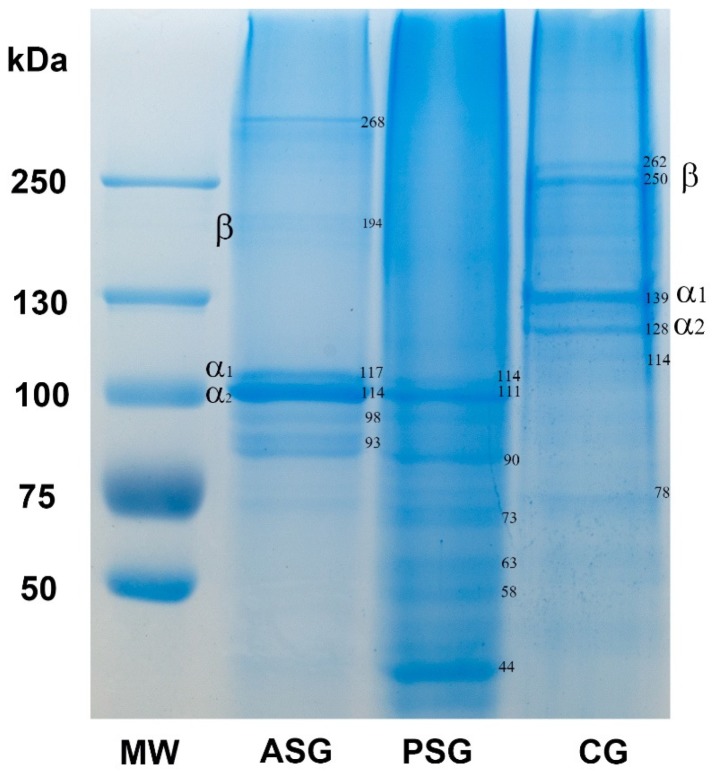
SDS-polyacrylamide gel electrophoresis (SDS-PAGE) showing ASG and PSG marine gelatins from *R. venosa* and CG from pig skin; MW—molecular weight marker. Numbers represent the molecular weight of different protein bands identified in marine and commercial gelatins.

Figure 1 shows the marine gelatins from *R. venosa* evaluated by electrophoresis in polyacrylamide gel and compared to that of CG from pig skin.

The ASG sample presented α- and β-chains, as major protein constituents, corresponding to the following molecular weights: ~114–117 and ~194 kDa, respectively. For ASG, a higher molecular weight protein of ~268 kDa and two lower molecular weight proteins of ~93–98 kDa were also observed. The presence of β-dimer and the protein of ~268 kDa indicated that ASG contains intermolecular crosslinks which have not been hydrolyzed during the extraction. The electrophoretic pattern of ASG was similar with those reported for gelatin extracted from marine snails *Hexaplex trunculus* and *Ficus variegate* [16,17]. The PSG did not display β-chains, but revealed the presence of α-chains at ~111–114 kDa and several protein bands with low molecular mass of ~90, 73, 63, 58, and 44 kDa. This mixture of polypeptides is probably the result of a high degree of hydrolysis due to the pepsin treatment for 24 h and the additional heat treatment at 98 °C. Previous studies showed that gelatin extracted from shark skin and African catfish exhibited an increase in shorter chain fragments and a decrease in the intensity of high molecular weight chains, findings which were consistent with our results [36,37]. Both gelatin samples obtained in this study exhibited bands corresponding to α-chains with a lower molecular mass (~110–117 kDa) than that of CG (123–139 kDa). The CG presented the typical α- (~123–139 kDa) and β-chains (~250–262 kDa) corresponding to collagen type I and several bands of polypeptides with molecular weight of 114 and 78 kDa. Similar results were reported for collagen extracted from different fish species, which consisted of two α_1_ and one α_2_ chains with slightly lower molecular weight than collagen type I from calf skin [38]. Collagen from small-spotted catshark also exhibited α subunits lower than 110 kDa [39]. The differences in the α- and β-chains position between species is probably related to the number of amino acids, which differ between marine and mammalian collagens [7].

#### 2.1.3. Fourier Transform Infrared (FTIR) Spectroscopy

**Figure 2:**
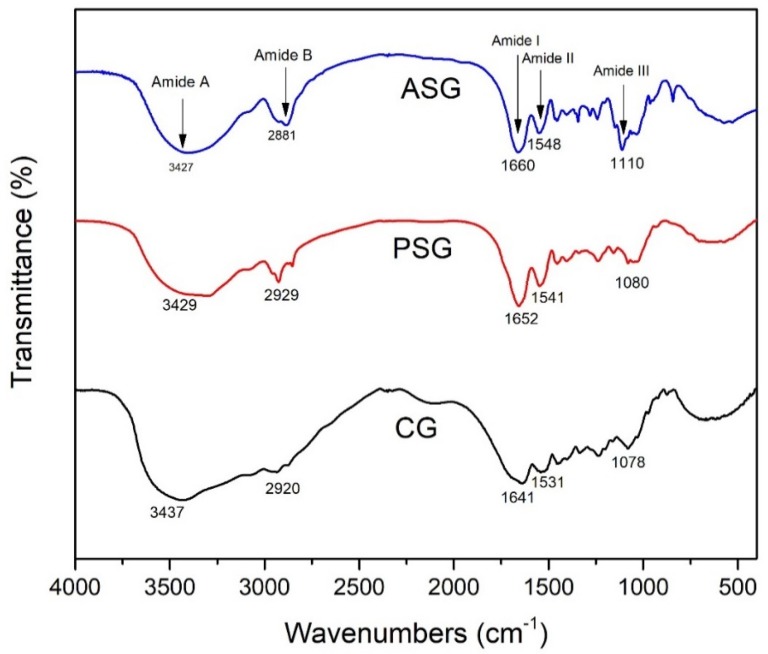
Fourier transform infrared (FTIR) spectra of ASG and PSG marine gelatins from *R. venosa* compared to CG from pig skin.

In order to study the secondary structure of gelatin samples isolated from marine snail, we have used FTIR spectroscopy analysis. The FTIR spectra of the tested samples are shown in Figure 2.

*R. venosa* gelatins exhibited main absorption bands, specific for the peptide bonds in the amide band regions. Thus, the amide A large band, associated with N–H stretching vibration, depending on the conformation of gelatin, shifted to lower wavenumbers for ASG (3427 cm^−1^) and PSG (3429 cm^−1^) samples compared to CG (3437 cm^−1^), which demonstrated a lower structural order of polypeptide chains. The amide B bands, corresponding to symmetric and asymmetric vibrations of C–H bonds, were observed at 2881 cm^−1^ for ASG. The amide I band corresponding to the C=O stretching vibration from amide group shifted towards higher wavenumbers, at 1660 and 1652 cm^−1^ for ASG and PSG, respectively, when compared to CG (1641 cm^−1^), due to the weaker H-bond formation in snail gelatins. The amide II band resulted from the overlap of amide N–H bending and C–N stretching vibrations appeared at 1548 cm^−1^ for ASG sample and shifted at 1541 cm^−1^ for PSG sample, probably as a result of higher content of imide bonds formation. The amide II band of CG from 1531 cm^−1^ was larger and shifted towards lower wavenumbers than that of marine gelatins, which was consistent with a more disordered helical structure with more imide bonds formation. In the FTIR spectra, the amide III band was assigned to NH bending at 1230 cm^−1^, while C–O stretching vibrations superimposed with C–N stretching vibrations were identified at 1110, 1080, and 1078 cm^−1^ for ASG, PSG and CG, respectively, suggesting a degree of glycosylation [40].

Overall, these results indicated a comparable structure and chemical composition of the gelatins obtained from *R. venosa* using acidic or enzymatic extraction, but a structure less associated by hydrogen bonding in the case of ASG. In addition, slight differences between marine and pig skin gelatins were observed probably due to variations in the sequence of amino acids. Similar FTIR spectra were reported for collagen/gelatin extracted from *C. reniformis* marine sponge [10].

#### 2.1.4. Scanning Electron Microscopy (SEM) Observations

**Figure 3:**
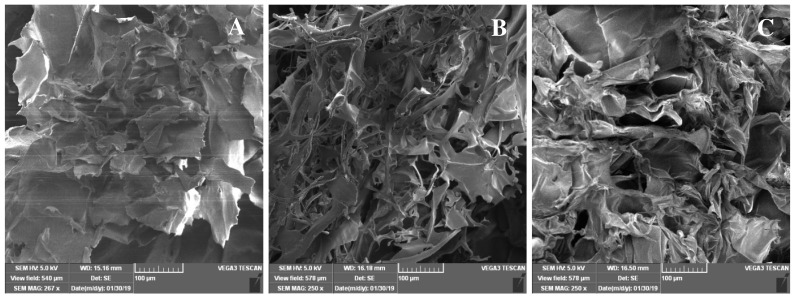
Scanning electron micrographs showing the surface of freeze-dried (**A**) ASG and (**B**) PSG from *R. venosa* and (**C**) CG from pig skin.

The ultrastructure of the obtained samples was observed by SEM. The lyophilized samples of ASG exhibited a rough, multilayered appearance, in contrast to PSG and CG samples, which showed a fibril-like network forming a microporous structure with uneven sized pores (Figure 3). A previous study has reported a nonfibrillar form of collagen/gelatin extract obtained from *C. reniformis* marine sponge, which presented a nodular structure with a rough appearance [10].

### 2.2. In Vitro Biocompatibility of Marine Gelatins

#### 2.2.1. Evaluation of Cell Viability

**Figure 4:**
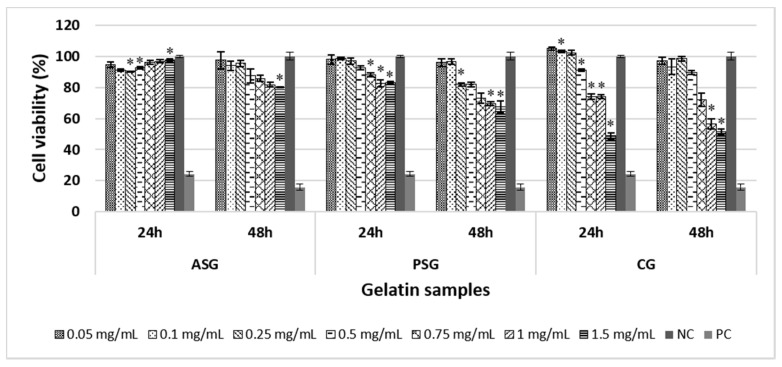
Cell viability of HaCaT cells exposed to increasing concentrations of ASG, PSG, and CG samples for 24 and 48 h, as evaluated by MTT assay. The negative control (NC) was represented by untreated cells and the positive control (PC) was represented by 100 μM H_2_O_2_. All samples were normalized to the NC considered to be 100% viable. Data were presented as mean ± SD (*n* = 3). * *p* < 0.05 compared to the NC.

The percentage of cell viability after the treatment with different concentrations of ASG, PSG, and CG was assessed by MTT assay, which evaluates the activity of mitochondrial dehydrogenases. ASG sample showed a good biocompatibility at all tested concentrations (0.05–1.5 mg/mL) and at both exposure times (24 and 48 h) (Figure 4). All cell viability values were above 80% (noncytotoxic effect), ranging between 90.49% and 97.59% after 24 h of treatment, and between 80.05% and 97.47% after 48 h. On the other hand, PSG sample showed no cytotoxic activity at all tested concentrations after 24 h of exposure (viability values between 82.55% and 98.68%), whereas CG exhibited viability values above 80% only within the concentration range of 0.05–0.5 mg/mL. After 48 h, PSG maintained a good biocompatibility up to the concentration of 0.5 mg/mL (viability values between 81.93% and 96.63%), but cell viability decreased below 75% at higher concentrations. The CG sample showed a similar profile with that observed after 24 h of treatment, with viability values below 80% at concentrations higher than 0.5 mg/mL. Overall, the ASG exhibited no cytotoxic effect at all tested concentrations, whereas the PSG and CG showed a similar biocompatibility, with cell viability values decreasing below 80% at concentrations higher than 0.5 mg/mL.

Previous studies have reported no cytotoxic effects of collagen/gelatin obtained from different marine sources on various cell lines. For example, collagen from codfish skin did not affect lung fibroblast metabolism at concentrations ranging between 0.01 and 0.05 mg/mL, but exhibited cytotoxic activity at concentrations higher than 0.1 mg/mL [41]. Collagen obtained from the starfish *Asterias amurensis* also promoted growth and viability of human dermal fibroblasts at concentrations ranging between 0.01 and 1 mg/mL [42], whereas squid gelatin peptides (0.025–0.1 mg/mL) exhibited a dose-dependent increase of cell viability in oxidation-induced human lung fibroblasts [43].

#### 2.2.2. Morphological Examination

**Figure 5:**
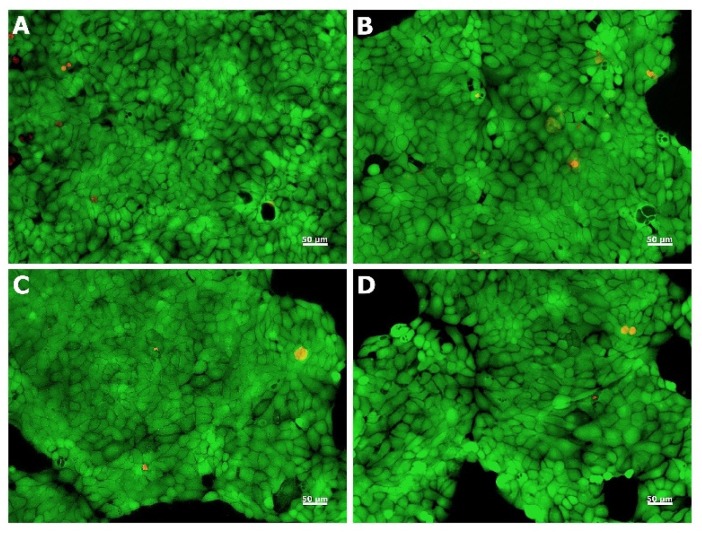
Live/dead staining with calcein-AM (green) and ethidium homodimer-1 (red) of HaCaT cells untreated control; (**A**) and treated with ASG (**B**), PSG (**C**), and CG (**D**) at the concentration of 0.25 mg/mL.

Live/dead staining was used to evaluate the cell morphology and viability after the treatment with marine gelatin samples. Thus, HaCaT cells maintained their viability after 48 h of cultivation in the presence of marine and commercial gelatins, while few dead cells could be observed (Figure 5).

Furthermore, cells treated with marine and commercial gelatins showed no significant morphological changes, maintaining their normal phenotype when compared to that of the untreated cells. In addition, quantitative analysis of cell density performed with ImageJ software showed that more than 99% of cells were viable for all samples. ASG, PSG, and CG promoted growth and viability of human keratinocytes, results which correlated well with those obtained by the quantitative MTT assay.

### 2.3. Biological Properties of Marine Gelatins

#### 2.3.1. Cell Adhesion Capacity

**Figure 6:**
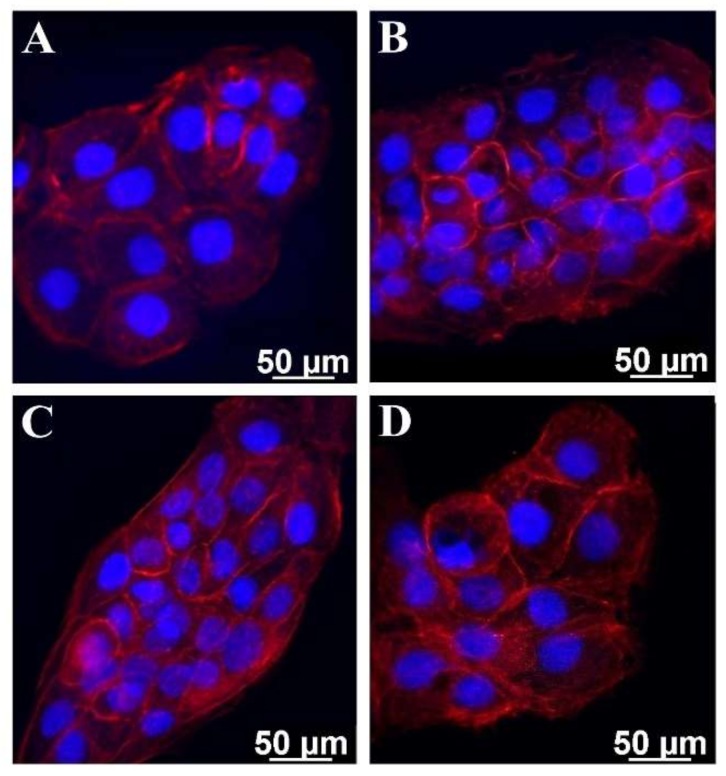
Distribution of actin filaments in HaCaT cells, assessed by fluorescence microscopy. HaCaT cells adhered to plastic (**A**) and to 0.25 mg/mL ASG (**B**), PSG (**C**), and CG coatings (**D**). Cells were stained for actin (red) and nuclei (blue).

The adhesion of HaCaT cells on gelatin coatings was evaluated using phalloidin TRITC staining, which highlighted the actin filaments. The distribution of actin revealed the morphological changes at the cytoskeleton level in HaCaT cells (Figure 6).

In the control, cells exhibited a polygonal-shaped morphology typical of HaCaT cells, with actin filaments assembled into large radial bundles (Figure 6A). The cells cultivated on ASG- and PSG-coated coverslips presented a different actin distribution, with smaller filaments which were visualized close to the nucleus (Figure 6B,C). The cells that adhered to the CG coating presented morphological features similar to the control (Figure 6A,D).

#### 2.3.2. Irritant Potential

According to the Organization for Economic Cooperation and Development (OECD) guidelines for testing of chemicals, a substance is considered to be an irritant if it has the ability to decrease cell viability below the defined threshold of 50%, when compared to the negative control (untreated cells). In our study, marine gelatin samples exhibited cell viability values higher than 50% at all tested concentrations, with values ranging between 73.33% and 108.33% and, therefore, can be considered non-irritants for skin. These results were also correlated with the low release of IL-6 and IL-1α proinflammatory cytokines as measured by ELISA assay. These interleukins are active and pleiotropic inflammatory cytokines that play a key role in the inflammatory process [44,45]. IL-1α is a known endpoint to predict skin irritation and the concentration of IL-1α released by keratinocytes in culture medium has been reported to increase after exposure to different irritants [46]. In our study, the cytokine content, expressed as pg/mL, varied with the sample concentration (Table 2 and Table 3). Thus, in the case of ASG, the IL-1α content varied from 1.39 to 6.72, whereas slightly higher values were observed for PSG (Table 2). For the CG sample, the concentration of IL-1α was similar to that of the negative control, ranging between 0.37 and 1.06. However, all these values were significantly lower than that of 0.1% SDS (40.93) used as positive control (Table 2).

The levels of IL-6 increased from 54.08 at the concentration of 0.1 mg/mL to 279.65 at the concentration of 0.75 mg/mL for ASG, and ranged between 92.67 and 384.02 for PSG (Table 3). Although the concentration of IL-6 released in culture medium was higher for PSG compared to ASG, all values were significantly lower compared to the positive control (1094.09). For the CG sample, the IL-6 content was slightly higher than that of the negative control, ranging between 30.49 and 98.52 (Table 3). For TNF-α, no evidence of the release of this cytokine was detected in the cell culture medium after the treatment with gelatin samples for 18 h. Similar results were reported by Alves et al. [47] in the case of human keratinocyte treatment with codfish skin collagen, where no release of IL-6 and IL-18 was detected in the culture medium, highlighting the non-irritant effect and cosmetic potential of marine collagen.

## 3. Experimental Section

### 3.1. Raw Materials

Marine snails were collected in August 2018 from the Romanian seacoast of the Black Sea between the 2 Mai and Vama Veche areas. The samples were washed with cold distilled water and stored at −20 °C until use. CG from pig skin and other chemicals were purchased from Sigma-Aldrich (Saint Louis, MO, USA) unless otherwise specified.

### 3.2. Gelatin Extraction

Snail soft tissue was removed from the hard shell, washed with distilled water for 30 min, and cut into small pieces (2–5 mm) using scissors. In order to remove noncollagenous proteins, the cleaned tissue was pretreated with 0.5 M NaOH solution in a ratio of 1:10 (*w*/*v*) at room temperature for 24 h. After centrifugation at 5000 *g* for 30 min, the obtained residue was washed with distilled water until neutral pH was achieved. Gelatin was extracted using acidic and enzymatic methods.

Acidic extraction was performed by gentle stirring of the pretreated tissue in 0.5 M acetic acid solution (1:10, *w*/*v*) at room temperature for 24 h. The sample was centrifuged at 8000 *g* for 40 min and heated at 60 °C in a shaking water bath (Witeg, Wertheim, Germany) for 20 h. Then, the ASG solution was filtered to remove the insoluble material, dialyzed against distilled water, and freeze-dried at −40 °C for 48 h.

For enzymatic extraction, the pretreated tissue was digested using pepsin from porcine gastric mucosa (2000 FIP-U/g, Carl Roth, Karlsruhe, Germany) in 0.5 M acetic acid solution at a pepsin/dry tissue ratio of 1:10 (*w*/*w*) and continuously stirred at room temperature for 24 h. The sample was centrifuged at 8000 *g* for 40 min and the resulting solution was subjected to thermal treatment at 98 °C for 1 min, in order to inactivate the enzyme. Then, the solution was heated at 60 °C in a shaking water bath for 20 h. Finally, the PSG solution was filtered, dialyzed against distilled water, and freeze-dried at −40 °C for 48 h. The obtained gelatin powders were stored at 4 °C until use.

### 3.3. Yield of Gelatin Extraction

The yield of gelatin extraction was calculated based on wet weight of fresh tissue using the following formula:

Extraction yield (%) = Gelatin dried weight/Fresh tissuewet weight × 100.

### 3.4. Protein Content

The total protein content was assessed using a bicinchoninic acid (BCA) protein assay kit, according to the manufacturer’s instructions. The absorbance of the samples was read at 562 nm, using an UV/VIS spectrophotometer (Jasco, V650, Tokyo, Japan). Bovine serum albumin (BSA) was used as standard.

### 3.5. Hydroxyproline Content

Hydroxyproline (Hyp) content of gelatin samples was determined according to the method of Edwards and O’Brien Jr [48], with several modifications. Briefly, gelatin samples (0.05 g) were hydrolyzed in 5 mL perchloric acid 70 % at 120 °C for 8 h. The solutions were neutralized with 2.5 N NaOH at pH 6 and then an oxidant solution (a mixture of 1.41% chloramine T and acetate/citrate buffer, pH 6) was added. The mixtures were incubated at room temperature for 20 min and, finally, 26% perchloric acid and 10% 4-(dimethylamino)benzaldehyde (DMAB) dissolved in *n*-propanol were added. The solutions were heated at 60 °C for 20 min and the absorbance was then read at 560 nm using an UV/VIS spectrophotometer (Jasco, V650, Tokyo, Japan). A hydroxyproline standard curve was prepared from serial dilutions in the range of concentrations 1–10 µg/mL. The Hyp content was expressed as g/100 g dry weight.

### 3.6. Differential Scanning Calorimetry

The melting temperature of gelatins was assessed by differential scanning calorimetry (DSC) using a Mettler Toledo (Greifensee, Switzerland) equipment. Freeze-dried samples (2–3 mg) were mixed with 10 µL distilled water and incubated at temperatures ranging between 25 and 85 °C, with a heating rate of 2.5 °C/min.

### 3.7. SDS-PAGE Analysis

SDS-PAGE was conducted according to the method of Laemmli [49] with minor modifications. Samples were diluted in Laemmli buffer at a ratio of 1:2 (*v*/*v*), heated at 100 °C for 5 min, and loaded on a 5% stacking gel and a 7.5% resolving gel. Then, they were migrated in a vertical gel unit (Biometra, Analytik Jena, Jena, Germany) at a constant current of 10 mA for 4 h. After electrophoresis, the gels were stained using Roti Blue solution (Carl-Roth, Karlsruhe, Germany), destained in a solution of methanol/distilled water 1:3, and photographed. Commercial gelatin (CG) from pig skin was used as control and high molecular weight marker (55–250 kDa) was migrated in the same conditions. The molecular weight of protein bands was determined using a logarithmic regression analysis by plotting the log of molecular weight versus relative mobility.

### 3.8. FTIR Spectroscopy

Gelatin samples were mixed with potassium bromide (KBr) and ground into powder. FTIR spectra were performed in the range of wavelength between 4000 and 400 cm^−1^ with a resolution of 5 cm^−1^ using a Bruker Tensor 27 (Billerica, MA, USA) infrared spectrometer. A total of 50 scans was carried out for each sample.

### 3.9. Scanning Electron Microscopy

The structural morphology of gelatins was examined by SEM. Lyophilized samples were coated with a gold layer and visualized on TESCAN VEGA 3 LMH scanning microscope (Brno, Czech Republic) operated at 15 kV in low vacuum mode.

### 3.10. Cell Viability Evaluation 

In vitro experiments were performed on the spontaneously immortalized human keratinocyte cell line HaCaT purchased from AddexBio, San Diego, CA, USA. Cells were grown in RPMI 1640 culture medium (Biochrom, Berlin, Germany) supplemented with 10% fetal bovine serum (FBS) and 1% antibiotics (penicillin, streptomycin, and neomycin) at 37 °C in a humidified atmosphere with 5% CO_2_. In order to evaluate cell cytotoxicity, HaCaT cells were seeded in 96-well culture plates at a density of 5 × 10^4^ cells/mL and incubated for 24 h to allow cell attachment. After this period, fresh medium containing different concentrations of ASG, PSG, and CG (0.05; 0.1; 0.25; 0.5; 0.75; 1 and 1.5 mg/mL) were added into each well and plates were incubated in standard conditions for 24 and 48 h, respectively. Untreated cells and cells cultivated in the presence of 100 μM H_2_O_2_ served as negative and positive controls, respectively. Cell metabolic activity was measured using an MTT assay [47]. Briefly, MTT working solution (0.25 mg/mL prepared in culture medium without FBS) was added to the cells and the plates were incubated at 37 °C for 3 h. The insoluble formazan crystals were dissolved using isopropanol and, after 15 min of incubation at room temperature with gentle stirring, the absorbance was read at 570 nm using a Mithras LB 940 microplate reader (Berthold Technologies, Bad Wildbad, Germany). The recorded value directly correlates to the number of metabolically active cells. The results of the MTT assay were calculated using the following equation: %cell viability = sample absorbance/negative control absorbance × 100. The negative control was considered 100% viable.

### 3.11. Live/Dead Assay

Cell morphology was assessed by fluorescence microscopy using the Live/Dead assay kit (Molecular Probes, Thermo Fisher Scientific, Eugene, OR, USA) according to the manufacturer’s instructions. Briefly, after 48 h of cultivation in the presence of different gelatin samples, cells were stained with calcein-AM (2 μM) and ethidium homodimer-1 (4 μM) at room temperature for 30 min. Fluorescent images were acquired using an Axio Observer D1 microscope and analyzed using AxioVision 4.6 software (Carl Zeiss, Oberkochen, Germany). All images were processed using ImageJ 1.51 software (Bethesda, MD, USA) and quantitative analysis of cell density were performed counting calcein and ethidium homodimer-1 positive cells. The obtained values have been normalized to the control (100% viability).

### 3.12. Cell Adhesion Assay

For cell adhesion assay, solutions containing 0.25 mg/mL of ASG, PSG and CG were added on coverslips previously inserted in 24-well plates and allowed to dry at room temperature, overnight. After evaporation, coatings were sterilized by UV irradiation for 3 h. HaCaT cells were seeded at a density of 1 × 10^5^ cells/mL on coverslips coated with gelatin solution. Coverslips without gelatin coatings were used as control. After 24 h of incubation at 37 °C in a humidified atmosphere with 5% CO_2_, the culture medium was discarded, and cells were washed with PBS and fixed with 4% formaldehyde. Cells were permeated with 0.1% Triton X-100 solution at room temperature for 10 min and stained with phalloidin TRITC (50 μg/mL) for 40 min and then with DAPI (1 μg/mL) for 10 min. Fluorescent images were acquired using an Axio Observer D1 microscope and analyzed using AxioVision 4.6 software (Carl Zeiss, Oberkochen, Germany). All images were processed using ImageJ 1.51 software (Bethesda, MD, USA).

### 3.13. Irritant Potential Test

In order to assess the irritant potential of gelatins, HaCaT cells were seeded in 24-well culture plates at a density of 1 × 10^5^ cells/mL and cultivated in RPMI 1640 medium supplemented with 10% FBS. After 24 h, gelatin samples (0.1; 0.25; 0.5, and 0.75 mg/mL), negative control (cells grown in culture medium) and positive control (cells treated with 0.1% SDS) were added to the cells and incubated in standard conditions for 18 h. The culture medium was collected and used for cytokine analysis. Levels of IL-6, IL-1α, and TNF-α proinflammatory cytokines were determined using commercial ELISA kits, according to the manufacturer’s instructions (Invitrogen, Thermo Fisher Scientific, Vienna, Austria). The absorbance was recorded at 450 nm using a microplate reader Tecan Sunrise (Tecan, Grodig, Austria).

### 3.14. Statistical Analysis

All experiments were performed in triplicate, and the data are presented as mean ± standard deviation (SD). Statistical analysis was performed using Student *t*-test. A value of *p* < 0.05 was considered to be significant.

## 4. Conclusions

Gelatin was isolated for the first time from the marine snail *R. venosa* using acidic and enzymatic methods. The samples presented comparable values to commercial gelatin, in terms of protein, hydroxyproline content, and melting temperature. The structural features highlighted by FTIR spectra were slightly different for all tested gelatins. The electrophoretic profile showed a high degree of hydrolysis and peptides with low molecular weight for the enzymatic gelatin compared to acidic gelatin. Regarding the in vitro tests, gelatin obtained by chemical treatment showed a better cytocompatibility compared to the enzymatic extracted gelatin, with no signs of cytotoxicity and irritant potential. In addition, both marine gelatins promoted cell proliferation and cell-induced adhesion capacity when compared to mammalian gelatin. Overall, our results suggested that *R. venosa* marine snail could be a valuable alternative and safe source of gelatin, useful as an additive in biomedical and pharmaceutical skincare products.

## Figures and Tables

**Table 1:** Yield and characteristics of acid-solubilized gelatin (ASG) and pepsin-solubilized gelatin (PSG) from *R. venosa* and commercial pig skin gelatin (CG). The results are expressed as mean ± SD (*n* = 3). * *p* < 0.05, compared to CG sample.

Gelatin Type	Extraction Yield (%)	Protein Content (%)	Hyp Content (%)	Melting Temperature (°C)
ASG	9.71 ± 0.38	91.48 ± 4.61	10.62 ± 0.37	35.30 ± 1.56
PSG	8.65 ± 0.42	83.12 ± 3.30	9.39 ± 0.51 *	33.20 ± 1.38
CG	-	86.12 ± 3.23	11.17 ± 0.21	28.80 ± 1.93

**Table 2:** IL-1α secretion levels expressed as pg/mL in the culture medium of HaCaT keratinocytes treated with increasing concentrations of marine gelatin samples.

Sample		Tested Concentrations			
		0.1 mg/mL	0.25 mg/mL	0.5 mg/mL	0.75 mg/mL
ASG		1.39 ± 0.27 *	1.10 ± 0.20 *	3.40 ± 0.55 *	6.72 ± 1.27 *
PSG		1.90 ± 0.87 *	2.82 ± 0.94 *	5.85 ± 1.83 *	7.35 ± 1.00 *
CG		0.37 ± 0.11 *	0.61 ± 0.18 *	0.77 ± 0.21 *	1.06 ± 0.13 *
NC	0.34 ± 0.07 *				
PC	40.93 ± 4.02				

NC—negative control (untreated cells), PC—positive control (SDS 0.1%). Values are expressed as mean ± SD (*n* = 3). * *p* < 0.05 compared to PC.

**Table 3:** IL-6 secretion levels expressed as pg/mL in the culture medium of HaCaT keratinocytes treated with increasing concentrations of marine gelatin samples.

Sample		Tested Concentrations			
		0.1 mg/mL	0.25 mg/mL	0.5 mg/mL	0.75 mg/mL
ASG		54.08 ± 1.49 *	162.81 ± 3.03 *	244.24 ± 19.70	279.65 ± 41.15 *
PSG		92.67 ± 7.55 *	196.44 ± 6.79 *	311.89 ± 16.10 *	384.02 ± 15.64
CG		46.46 ± 3.10 *	30.49 ± 1.59 *	98.52 ± 1.78 *	42.53 ± 4.65 *
NC	6.45 ± 2.30 *				
PC	1094.88 ± 188.96				

NC—negative control (untreated cells), PC—positive control (SDS 0.1%). Values are expressed as mean ± SD (*n* = 3). * *p* < 0.05 compared to PC.

## Supplementary Materials

The following are available online at https://www.mdpi.com/1660-3397/17/10/589/s1, Figure S1: Thermal melting curves of ASG and PSG from *R. venosa* and CG from pig skin.
