# Supplementary material for: Physicochemical and Biological Properties of Gelatin Extracted from Marine Snail Rapana venosa
Source: Mar Drugs. 2019 Oct 17;17(10):589. doi: 10.3390/md17100589 (PMC6835507; doi:10.3390/md17100589)
Supplement: Supplementary file 1 [file marinedrugs-17-00589-s001.zip › marinedrugs-570453-SI.pdf]

Supplementary Material

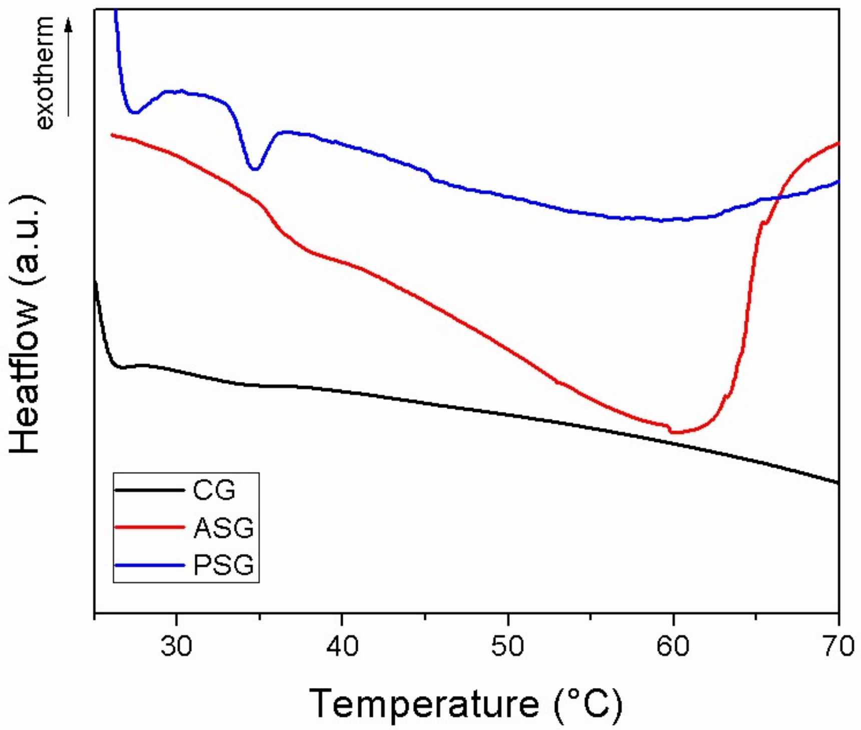

Figure S1. Thermal melting curves of ASG and PSG marine gelatins from *R. venosa* and CG from pig skin.
